# Deep-Sequencing Analysis of the Mouse Transcriptome Response to Infection with *Brucella melitensis* Strains of Differing Virulence

**DOI:** 10.1371/journal.pone.0028485

**Published:** 2011-12-28

**Authors:** Fangkun Wang, Sen Hu, Wenxing Liu, Zujian Qiao, Yuzhe Gao, Zhigao Bu

**Affiliations:** 1 State Key Laboratory of Veterinary Biotechnology and Zoonosis Laboratory of the Ministry of Agriculture, Harbin Veterinary Research Institute, Chinese Academy of Agricultural Sciences, Harbin, Heilongjiang, People's Republic of China; 2 Department of Preventive Veterinary Medicine, College of Animal Science and Veterinary Medicine, Shandong Agricultural University, Tai An, Shandong, People's Republic of China; University of North Carolina at Charlotte, United States of America

## Abstract

*Brucella melitensis* is an important zoonotic pathogen that causes brucellosis, a disease that affects sheep, cattle and occasionally humans. *B. melitensis* strain M5-90, a live attenuated vaccine cultured from *B. melitensis* strain M28, has been used as an effective tool in the control of brucellosis in goats and sheep in China. However, the molecular changes leading to attenuated virulence and pathogenicity in *B. melitensis* remain poorly understood. In this study we employed the Illumina Genome Analyzer platform to perform genome-wide digital gene expression (DGE) analysis of mouse peritoneal macrophage responses to *B. melitensis* infection. Many parallel changes in gene expression profiles were observed in M28- and M5-90-infected macrophages, suggesting that they employ similar survival strategies, notably the induction of anti-inflammatory and antiapoptotic factors. Moreover, 1019 differentially expressed macrophage transcripts were identified 4 h after infection with the different *B. melitensis* strains, and these differential transcripts notably identified genes involved in the lysosome and mitogen-activated protein kinase (MAPK) pathways. Further analysis employed gene ontology (GO) analysis: high-enrichment GOs identified endocytosis, inflammatory, apoptosis, and transport pathways. Path-Net and Signal-Net analysis highlighted the MAPK pathway as the key regulatory pathway. Moreover, the key differentially expressed genes of the significant pathways were apoptosis-related. These findings demonstrate previously unrecognized changes in gene transcription that are associated with *B. melitensis* infection of macrophages, and the central signaling pathways identified here merit further investigation. Our data provide new insights into the molecular attenuation mechanism of strain M5-90 and will facilitate the generation of new attenuated vaccine strains with enhanced efficacy.

## Introduction

*Brucella* spp are facultative intracellular, Gram-negative coccobacilli belonging to the alpha subclass of Proteobacteria. They reside in professional and non-professional phagocytes, and macrophages in particular are a major target in infected mammals. In contrast to other pathogenic bacteria, *Brucella* spp appear to lack classical virulence factors such as exotoxins, capsules, fimbriae, invasive proteases, virulence plasmids and lysogenic phages, and their virulence is instead associated with their capacity to invade and propagate within host cells [1]. Intracellular survival and multiplication of *Brucella* is thought to depend crucially on avoidance of fusion with late endosomes and lysosomes [2]. *Brucella melitensis*, the first species described in the genus *Brucella*, causes abortions in goats and sheep and Malta fever in human. In humans, *B. melitensis* is highly pathogenic, making it one of the most serious zoonoses in the world. To date, several strategies have been employed to develop vaccines against brucellosis, but no satisfactory vaccine has been discovered to combat the disease in humans, and animal vaccines are pathogenic to humans [3]. Because of its epidemic potential, the effectiveness of aerosol infection, the absence of a human vaccine, and the drawbacks of current vaccine strains in terms of safety, this agent has been classified as a biosafety level 3 pathogen and is considered to be a potential bioterrorism agent [4]. The attenuated *B. melitensis* vaccine smooth strain M5-90 was derived from the virulent smooth *B. melitensis* M28 ovine isolate by serial passage in chicken, acriflavine treatment, and further passage for 90 generations in chicken embryo fibroblasts [5]. This attenuated live vaccine is considered to be one of the key factors that caused a rapid decline in the incidence of brucellosis in animals and humans in China. The reduced virulence of the vaccine appears to be stable and the vaccine strain maintains its original biochemical and immunological characteristics. Nevertheless, the underlying molecular or physiological changes underlying loss of virulence are not understood.

Next-generation high-throughput deep-sequencing technology, such as digital gene expression tag profiling (DGE), has recently been adapted for transcriptome analysis [6], [7], [8]. This direct sequencing methodology allows for the identification of millions of short RNAs in a sample, and of differentially expressed genes (DEGs), without the need for prior annotations [9], [10], [11]. Sequencing-based methods generate absolute gene expression measurements and avoid many of the inherent limitations of earlier microarray-based assays [12], [13], [14].

In many tissues macrophages constitute the first line of defense of the innate immune response against invading microorganisms [15]. One study provided evidence that virulent *Brucella abortus* strain 2308 survives at significantly higher levels in normal mouse peritoneal macrophages and in macrophages treated with gamma interferon than does the attenuated strain 19 [16]. Although the transcriptional profiles of *Brucella*-infected mouse macrophages have been studied previously [17], [18], [19], no studies have compared host responses between infections with a virulent *B. melitensis* strain and its attenuated vaccine strain. In the present study we employed the Illumina Genome Analyzer platform to perform DGE analysis of changes in the peritoneal macrophage transcriptome in response to *B. melitensis* infection. Macrophage gene expression profiles after *B. melitensis* infection were systematically analyzed, and both common and strain-specific transcriptional responses to *B. melitensis* infection were evaluated. The findings reported here cast new light both on the molecular interactions between *Brucella* and its host and on the changes that underpin the attenuation of strain M5-90. These data provide a firm foundation for further work aimed at reducing the residual virulence of the vaccine strain and thereby enhancing vaccine efficacy.

## Materials and Methods

### Ethics statement and animal experimentation

Care of laboratory animals and animal experimentation were preformed in accordance with the Beijing Administration Guidelines for the Use of Laboratory Animals. All animal studies were approved by the Review Board of Harbin Veterinary Research Institute and by the Animal Care and Use Committee of Heilongjiang Province (SYXK(H)2006-032). Animal studies with *Brucella* infected macrophages were conducted in a biosecurity level 3+ laboratory approved by the Chinese Ministry of Agriculture.

### Culture of bacterial strains and mouse peritoneal macrophages

*B. melitensis* strain M28 and the attenuated strain M5-90 (laboratory stock) were grown on Tryptic Soy Broth (TSB) plates containing 1% agar under biosecurity level 3+ conditions. After 2 days at 37°C, cells were collected by scraping and were suspended in phosphate-buffered saline (PBS; pH 7.2). Colony forming units (CFUs) were determined by plating on agar and incubating for 3 days at 37°C under 5% CO_2_.

BALB/c female mice aged 6–8 weeks were purchased from Vital River Laboratories (VRL, Beijing, China). Peritoneal macrophages were recovered by peritoneal lavage with 5 ml of cold RPMI 1640 medium (Invitrogen, Carlsbad, CA, USA) and isolated by centrifugation according to the method described by Kim [20] with some modifications. For adherent cells, cells (2×10^6^) were plated on six-well tissue culture plates and cultured in RPMI 1640 medium supplemented with 10% fetal bovine serum (FBS), 0.2 mM L-glutamine, antibiotic–antimycotic (100 U/ml penicillin G; 100 µg/ml streptomycin) for 2 h in a humidified incubator at 37°C under 5% CO_2_. Non-adherent cells were removed by washing once with RPMI 1640 medium containing 10% FBS, and then washed twice with Hanks' balanced salt solution. Adherent cells were cultured in RPMI 1640 medium with 10% FBS in an atmosphere containing 5% CO_2_.

### Macrophage infection and RNA isolation

Peritoneal macrophages from 10 mice were pooled and plated at 2×10^6^ cells/well in six-well tissue-culture plates in supplemented RPMI 1640 medium without antibiotics. After 12–24 h, macrophages were infected with 1 ml of stationary phase *Brucella* spp culture [multiplicity of infection (MOI) 200∶1]. Control macrophages and infected macrophages (M28 or M5-90) were incubated for a further 4 h at 37°C under 5% CO_2_, washed once with PBS, and then lysed for RNA purification (RNeasy, Qiagen, Germantown, MD, USA). All samples were quantified and examined for protein and reagent contamination by a Nanodrop ND-1000 spectrophotometer. RNA samples for analysis were selected based on a 28S/18S rRNA band intensity of 2∶1, a spectroscopic A260/A280 nm ratio of 1.8–2.0, and an A260/A230 nm ratio greater than 1.5.

### Digital gene expression tag profiling (DGE)

Reagents for DGE analysis were primarily from the Illumina (San Diego, CA, USA) Gene Expression Sample Preparation Kit and the Illumina Sequencing Chip (Flowcell); instrumentation was based on the Illumina Cluster Station and the Illumina HiSeq™ 2000 System. Sequence tag preparation was performed using the Digital Gene Expression Tag Profiling Kit (Illumina) according to the manufacturer's protocol. DNA was purified and subjected to Illumina sequencing. Image analysis, base calling and quality calibration were performed using the Solexa Automated Pipeline, after which the raw data (tag sequences and counts) were deposited in the GEO (http://www.ncbi.nlm.nih.gov/geo/) database under submission number GSE26337. Raw sequence reads were filtered by the Illumina pipeline. For analysis, adaptor tags, low-quality tags and tags with a copy number of one were excluded, and the clean tags generated were mapped to the reference sequences in the mouse genome (mm9 version from UCSC site) and the mouse transcriptome (all Refseq, mRNA and ESTs found in the GenBank database) with the criterion that only a single mismatch with the reference sequence was permitted. Tags that mapped to reference sequences of more than one transcript were excluded from analysis [12], [21].

### Screening of differentially expressed genes (DEGs)

To compare DEGs across samples (M28/Mc, M5-90/Mc, and M5-90/M28), the number of raw clean tags in each library was normalized to the number of tags per million (TPM) to obtain normalized gene expression levels. The detection of DEGs or tags across samples was performed as previously described [22], and the false discovery rate (FDR) was set below 0.01 [23]. FDR ≤0.001 and an absolute value of log2Ratio ≥1 was set as the threshold for significant gene expression differences.

### Cluster analysis of the DEG profiles

Genes with similar expression patterns often subserve overlapping functions. Accordingly, we performed cluster analysis of gene expression patterns using Cluster [24] and Java Treeview [25] software.

### Gene ontology (GO) functional enrichment analysis for DEGs

In gene expression profiling analysis, GO enrichment analysis of functional significance applies two-sided Fisher's exact test and χ^2^ testing to map all DEGs to terms in the GO database, searching for significantly enriched GO terms in DEGs compared to the genomic background. The FDR was calculated to correct the *P* value. We chose only GOs with a *P* value of <0.001 and a FDR of <0.05. Within the significant category, the enrichment factor Re was given by:
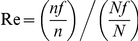
where nf is the number of DEGs within the particular category, n is the total number of genes within the same category, Nf is the number of DEGs in the gene reference database list, and N is the total number of genes in the gene reference database list.

### Pathway enrichment analysis for DEGs

Genes involved in different steps of a common pathway tend to overlap in their expression profiles, and pathway-based analysis can therefore cast light on biological function. Accordingly, further analysis consulted the Kyoto Encyclopedia of Genes and Genomes (KEGG), the major public pathway-related database. Pathway enrichment analysis identifies significantly enriched metabolic pathways or signal transduction pathways in DEGs versus the genomic background according to the formula:
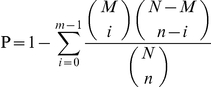
where N is the number of genes with KEGG annotation, n is the number of DEGs in N, M is the number of genes annotated to specific pathways, and m is the number of DEGs in M. Enrichment analysis of DEG pathway significance highlights the most meaningful pathways. Path-Net was the interaction net of the significant pathways of the DEGs (*P*<0.05), and was built according to the interactions among pathways of the KEGG database to reveal potential interactions between significant pathways [26]. Pathways that were statistically significant, but which markedly increased pathway bifurcation, thereby proving uninformative at the network level, were excluded from further analysis.

### Protein–protein interaction network analysis

Direct binding between different proteins can also reveal shared functionality. Protein–protein interaction network analysis integrates several important interaction network databases, including BIND(http://www.isc.org/software/bind), BioGrid(http://thebiogrid.org/) and HPRD(http://www.hprd.org/), and constructs interaction networks for protein-coding DEGs. Signal-net, a network map of differentially expressed proteins, was constructed using the KEGG interaction database. In Signal-net each cycle represents a gene. As an example, if there is evidence that two proteins interact with each other, an interaction edge is assigned between the two proteins where arrows or smooth edges indicate activation or inhibition [27], [28], [29], [30], [31].

### Quantitative PCR analysis

cDNA was synthesized using the Transcriptor First Strand cDNA Synthesis Kit (Roche, Indianapolis, USA). Quantitative PCR (qPCR) was then performed using the same RNA samples employed for DGE experiments on a Lightcycler480 (Roche), with SYBR Premix Ex Taq II (TaKaRa, Dalian, China). Each cDNA was analyzed in triplicate, after which the average threshold cycle (Ct) was calculated per sample. The relative expression levels were calculated with the 2^−ΔΔCt^ method; the average Ct value for all genes was used to correct for differences in cDNA input (Δ_Ct_ is the difference in Ct between the control Fnip1 products and the target gene products).

### Macrophage proteomic analysis

Protein samples were prepared from the cells in the same way as for the DGE experiments. After 4 h of incubation, infected macrophages were washed (250 mM sucrose, 10 mM Tris–HCl pH 7.0) and lysed in cold 0.2% v/v Triton X-100. Large cellular debris and contaminating bacteria were removed by low speed centrifugation (60 *g*, 5 min, 4°C). Supernatants were then centrifuged at 20,000 *g* for 1 h; pellets were resuspended in 1 ml of lysis buffer (7 M urea, 2 M thiourea, 4% w/v CHAPS) containing a protease inhibitor mix (GE Healthcare) and sonicated for 10 min on ice using a Sonifier 750 (Branson Ultrasonics, Danbury, CT, USA) with the following parameters: 2 s of sonication with a 6 s interval, and a 35% duty cycle. After the addition of nuclear mix (GE Healthcare), cell lysates was incubated for 1 h at 15°C to solubilize proteins. After centrifugation to remove insoluble material the supernatants were collected; protein concentrations were measured using the 2D Quant Kit (GE Healthcare) and 100 µg protein aliquots were stored at −74°C.

Sample labeling and 2D gel electrophoresis were performed according to previously published methods [32]. Immobilized pH gradient (IPG) strips (24 cm, pH range 4–7) (GE Healthcare) were used for isoelectric focusing (IEF). Vertical slab SDS–PAGE (12.5%) was run at 15 W/gel in a Ettan DAL twelve large vertical system (GE Healthcare). Gels were scanned using the Typhoon 9400 gel imager (GE Healthcare). Gel image analysis was performed using the DeCyder software suite (GE Healthcare) for image overlay and image analysis to identify alterations in protein spot intensities. Differentially expressed proteins were identified by MALDI-TOF-TOF [33].

## Results

### Analysis of DGE libraries

We investigated molecular changes taking place in mouse peritoneal macrophages following infection with virulent and vaccine strains of *Brucella*. Global gene expression profiles were analyzed using the Solexa/Illumina DGE system, a tag-based transcriptome sequencing method. cDNA libraries from uninfected macrophages (Mc) and from macrophages infected with strains M28 or M5-90 were sequenced using massively parallel sequencing on the Illumina platform. The major characteristics of these three libraries are summarized in Table 1.

**Table 1:** Categorization and abundance of tags.

Summary	Mc		M28		M5-90	
	Total	Distinct Tag^b^	Total	Distinct Tag	Total	Distinct Tag
Raw Data	3759000	194822	3664501	172507	3727501	185931
Clean Tag^a^	3735495	186252	3641485	165030	3704407	177764
All Tag Mapping to Gene	3243575	109441	3080483	93203	3113577	101527
Unambiguous Tag Mapping to Gene^c^	2367590	90978	2314976	77155	2341510	84452
Mapping to Mitochondrion	56546	475	40845	371	46324	408
Mapping to Genome	239715	41484	249901	37251	260929	41459
Unknown Tag	195659	34852	270256	34205	283577	34370

a Clean tags are the remaining tags after the filtering out of low-quality tags from the raw data.

b Distinct tags are different tag types.

c Unambiguous tags are the remaining clean tags after removal of the tags mapped to reference sequences from multiple genes.

We obtained approximately 3.7 million total sequence tags per library, and 184,420 distinct tag sequences. We filtered out adaptor tags, low-quality tags and tags with a copy number of one, generating ∼3.7 million clean sequence tags per library and 176,348 distinct clean tag sequences. Saturation analysis of the libraries revealed that the number of detected genes reduced progressively as the total number of sequence tags analyzed was increased, and the number of genes detected plateaued in each case when the number of sequence tags reached ∼2 million (Figure S1). Heterogeneity and redundancy are two significant characteristics of mRNA expression. To analyze the depth of the distribution of the ratio of distinct tag copy numbers between the two libraries, we determined that the number of distinct tags within five was approximately 99.05% of the total distinct tags (Figure S2).

### Analysis of tag mapping

For tag mapping we preprocessed one reference tag database that included 33,642 sequences from the *Mus musculus* RefSeq database. To obtain the reference tags we used *Nla*III to digest the samples; all CATG+17 tags in each gene were employed as reference tags for that gene. This generated a total of 303,456 reference tag sequences comprising 268,495 unambiguous tag sequences. In view of potential polymorphisms across samples, clean tags that mapped to reference sequences from multiple genes were filtered and tolerances were set to allow for a maximum of one mismatch. Using these criteria, 56.48–58.76% of the distinct clean tags mapped to the RefSeq virtual tag database, 46.75–48.85% of the distinct clean tags mapped unambiguously to the RefSeq virtual tag database, whereas 18.71–20.73% of the distinct clean tags did not map to this database (Table 1).

**Figure 1:**
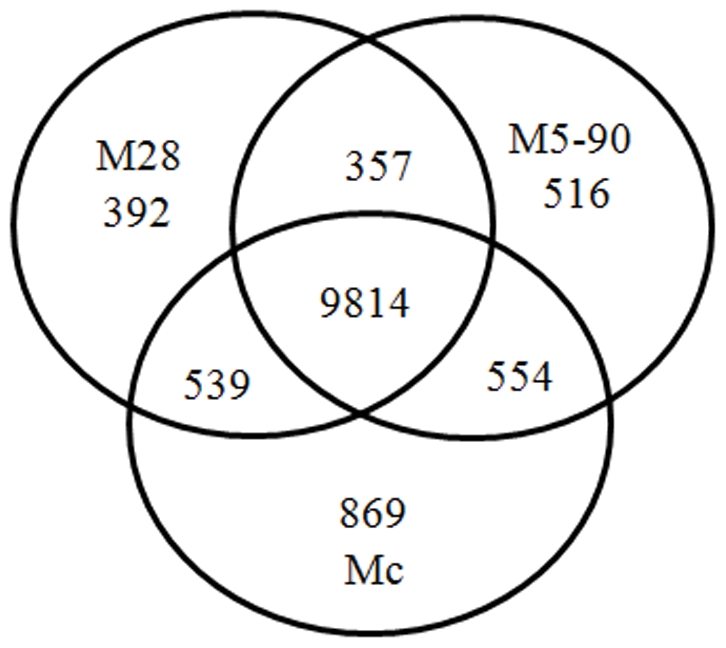
Comparative analysis of tag-mapped transcripts in the three libraries. A Venn diagram was created to quantify the overlap in the response. 10,171 transcripts were identified in both libraries, and 392 and 516 transcripts were present in the M28 and M5-90 libraries, respectively.

To determine the gene expression profiles we first mapped the unique tags from each library to the existing gene sequences. Analysis of the three libraries revealed 11,776, 11102 and 11241 tag-mapped sense transcripts for the Mc, M28 (virulent) and M5-90 (attenuated) libraries, respectively (Supplemental Table S1). To quantify the overlap in the response to infection a Venn diagram was created; 10,171 transcripts were present both libraries whereas 392 and 516 were present only in M28 or M5-90 macrophage-infected cells, respectively (Figure 1). Antisense genes can play an important role in gene expression; approximately 13.49, 13.48 and 12.72% of the transcripts identified for Mc, M28 and M5-90, respectively, were perfect matches for the antisense strand of known genes (Supplemental Table S2).

### Identification of DEGs

**Figure 2:**
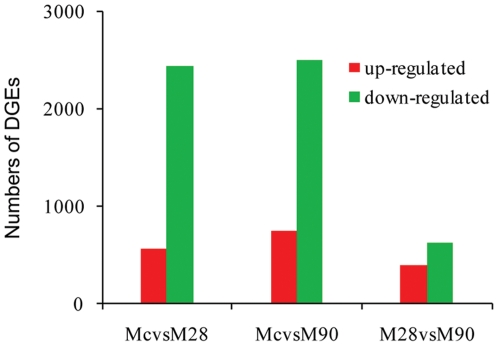
Differentially expressed genes between libraries. All genes mapped to the genome were examined for expression differences between the different libraries. Numbers of differentially expressed genes represent across sense transcripts, using threshold values FDR≤0.001 and log2 Ratio≥1 for controlling false discovery rates.

To gain insight into the global transcriptional changes taking place in *Brucella*-infected macrophages we applied a previously described method [22] to identify DEGs from the normalized DGE data by pairwise comparisons between samples (Mc versus M28, Mc versus M5-90, M28 versus M5-90). This identified 4353 genes with *P* values <0.0005, a false discovery rate (FDR) of <0.001, and estimated absolute log2 changes >1 in at least one of the pairwise comparisons (Figure 2). Statistical analysis confirmed the identification of 3008, 3247 and 1019 genes differentially transcribed in Mc versus M28, Mc versus M5-90 and M28 versus M5-90, respectively (Supplemental Table S3). Common changes in DEGs were observed between M28 and M5-90-infected macrophages, suggesting that similar gene expression changes take place in response to infection with virulent and vaccine strains of *Brucella*. Overlapping DEGs between the M28 and M5-90 libraries are likely to provide an insight into the molecular events related to different virulent *Brucella* infections.

Similar expression patterns can be indicative of overlapping biological function. We therefore performed cluster analysis of gene expression patterns using Cluster [24] and Java Treeview [25] software. The intersections of DEGs between Mc versus M28, Mc versus M5-90 and M28 versus M5-90 were clustered. We further analyzed the intersection of DEGs and found that 44 out of 187 library pairs that were upregulated in Mc versus M28, Mc versus M5-90 and M28 versus M5-90, and 38 were downregulated in Mc versus M28 and Mc versus M5-90, but upregulated in the M28 versus M5-90 library. Relative abundances were expressed as the TPM (transcripts per million mapped reads) Mc∶M28 (M5-90) or M28∶M5-90 ratios. As shown in Supplemental Table S4, the majority of the DEGs at the intersections between the two libraries identified genes, such as Acp5, LIPA and Atp6v0d2, that are implicated in lysosomal pathways. Some immune-related proteins (e.g. Cxcr4 and Ap2a2) involved in endocytosis were also detected. In addition, the analysis identified amino sugar and nucleotide sugar metabolism proteins (e.g. Cmas, Gfpt1 and Gnpda1), receptors related to cytokine–cytokine receptor interactions (Ccl9, Cxcr4, Vegfa and Il10ra), and other proteins, as detailed in Supplemental Table S4.

### Gene ontology (GO) enrichment analysis for DEGs (M28 versus M5-90 libraries)

**Figure 3:**
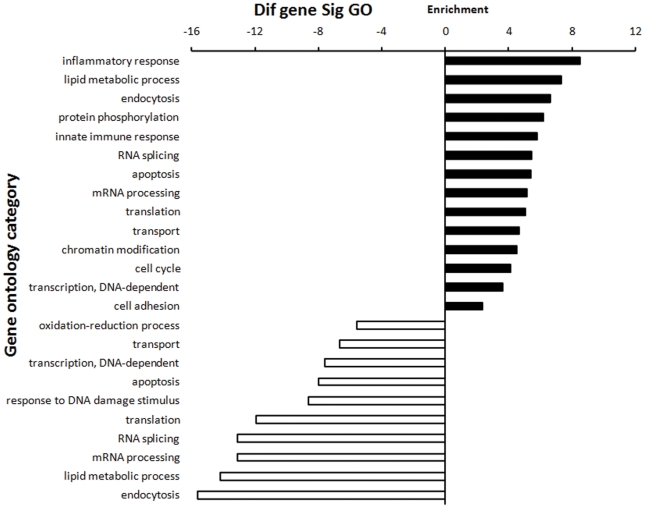
DGE (M28 versus M5-90 libraries) GO enrichment chart. The upper chart comprises GOs targeted by overexpressed DEGs; the lower chart comprises GOs targeted by downregulated DEGs. All these GOs show increased enrichment. The vertical axis is the GO category and the horizontal axis is the enrichment of GO.

To characterize the functional consequences of gene expression changes associated with infection with different virulent *Brucella* strains we performed GO enrichment analysis of DEGs based on the GO database; statistical significance was evaluated by two-sided Fisher's exact test and χ^2^ testing. We focused on GOs with a *P* value of <0.001 and a FDR of <0.05. As shown in Figure 3, high-enrichment GOs included the inflammatory response, lipid metabolism, endocytosis, the innate immune response, apoptosis, mRNA processing, transport, and cell adhesion. Of these, the maximum-enriched GO targeted by overexpressed DEGs was the inflammatory response. Among these inflammatory response-related genes, Ly96, Tlr1, Tlr7, and Tollip belong to Toll-like receptor signaling pathway. By contrast, the maximum-enriched GO for underexpressed DEGs was endocytosis (Ankfy1, Ap1s1, Ap3b1,Nostrin, and Usp33). Of these high-enrichment GOs, the transport GO (including proton, ion, mRNA, protein, and vesicle-mediated transport) contained the most DEGs identified from the M28 versus M5-90 library comparison. Among these transport-related genes, Abca2, Ap1s1, Ap3b1, Ap4s1, Atp6ap1, Mcoln1, Atp6v0b, and Atp6v0d2 belong to the lysosomal pathway; Stx3, Stx6,Vti1b, and Bet1l, belong to SNARE interactions in vesicular transport pathway.

### Pathway enrichment analysis for DEGs (M28 versus M5-90 libraries)

To characterize the functional consequences of gene expression changes associated with *Brucella* infection we performed pathway analysis of DEGs based on the KEGG database using the two-sided Fisher's exact test (Supplemental Table S5). Significant signaling pathways included the lysosomal, endocytosis, NOD-like receptor signaling pathway, Toll-like receptor signaling pathway, fatty acid metabolism, chemokine signaling pathway and basic metabolic pathways. The overwhelming majority of DEGs annotated in the lysosome pathway were downregulated, with the exception of Slc11a2 (formerly known as Nramp 2), one of the minor lysosome membrane proteins overexpressed in both infection libraries.

**Figure 4:**
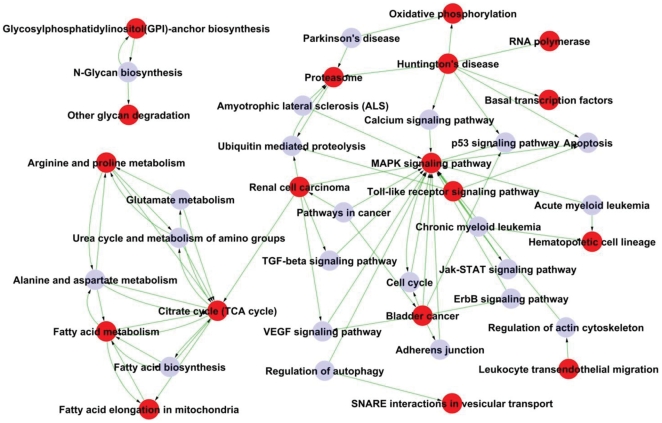
Path-Net of the significant pathways in the M28 versus M5-90 libraries (*P*<0.05). 18 out of the 29 significant pathways were chosen to build the Path-Net; potential interactions between the pathways were evaluated using Degree. Red dots represent significant pathways, grey dots represent other relevant pathways. The lines represent the interaction of the pathways.

As shown in Figure 4, Path-Net represents the interaction network of the significant pathways of M28 versus M5-90 (*P*<0.05) and was built according to interactions between KEGG database pathways of the to permit systematic identification of interactions between significant pathways identified in *Brucella*-infected macrophages [26]. We chose 29 significant pathways in Path-Net using ‘Degree’ to evaluate the pathway interactions. The significant pathways (degree≥5) are listed in Table 2. Significant pathway interactions in Path-Net were identified between the energy metabolism network (centered on the TCA cycle) and the cell apoptosis signal transduction network (centered on the MAPK signaling pathway). Associated networks included the renal cell carcinoma network and the citrate cycle pathway. In addition, a small net was evident that included three pathways involved in glycan metabolism.

**Table 2:** The significant pathways (Degree≥5) of Path-Net.

Pathway	Significant pathway	Indegree^a^	Outdegree^b^	Degree^c^
MAPK signaling pathway	YES	16	3	19
Citrate cycle (TCA cycle)	YES	7	6	13
Fatty acid metabolism	YES	4	4	8
Bladder cancer	YES	1	6	7
Huntington's disease	YES	0	7	7
Arginine and proline metabolism	YES	3	3	6
Renal cell carcinoma	YES	1	5	6
Alanine and aspartate metabolism		3	3	6
Proteasome	YES	4	1	5

a,b Indegree and Outdegree represent the inward and outward relationships, respectively;

c Degree evaluates the inferred extent of pathway interaction.

### Protein–protein interaction network analysis of the M28 versus M5-90 libraries

**Figure 5:**
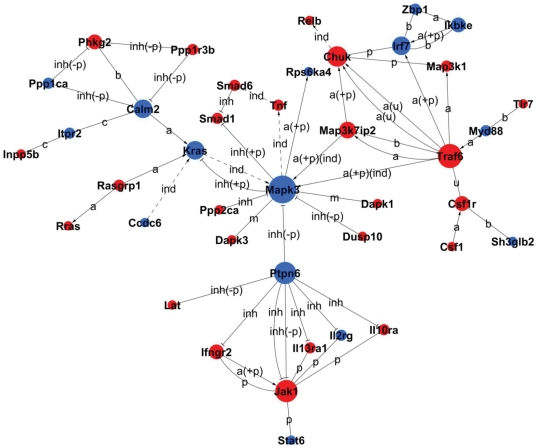
Signal-Net of the DEGs of the significant pathways of the M28 versus M5-90 libraries (*P*<0.05). Red and grey dots represent up- and downregulated genes, respectively; area sizes represent Degree, and lines represent interactions between the gene products.

Protein–protein interaction network analysis integrates several interaction network databases including BIND, BioGrid and HPRD, and constructs interaction networks for protein-coding DEGs. Betweenness Centrality represents a gene's mediate ability, and Degree represents the gene–gene interaction number. Indegree and Outdegree represent the upstream and downstream functions in the pathway [27], [28], [29], [30], [31]. Key genes with Degree≥5 are listed in Table 3; these include apoptosis-related genes such as Mapk3, Traf6, Chuk and Kras that participate in the MAPK signaling pathway, and also Jak1 and Ptpn6, key factors in the JAK–SAT signaling pathway that acts upstream of the MAPK signaling pathway (Figure 5).

**Table 3:** Key genes (Degree≥5) of Signal-Net.

Gene	Gene product	Expression	Betweenness centrality^a^	Degree	In^b^	Out^c^
Mapk3	mitogen-activated protein kinase 3	down	2.28E–02	12	8	6
Traf6	TNF receptor-associated factor 6	up	7.62E–03	9	2	8
Ptpn6	protein tyrosine phosphatase, non-receptor type 6	down	0	8	0	8
Jak1	Janus kinase 1	up	9.90E–04	8	7	1
Calm2	calmodulin 2	down	9.50E–03	7	6	5
Chuk	conserved helix-loop-helix ubiquitous kinase	up	1.88E–03	6	4	2
Itgb2	integrin beta 2	up	1.98E–04	5	4	2
Kras	v-Ki-ras2 Kirsten rat sarcoma viral oncogene homolog	down	1.11E–02	5	4	1
Pip5k1c	phosphatidylinositol-4-phosphate 5-kinase, type 1 gamma	up	1.39E–03	5	3	5
Irf7	interferon regulatory factor 7	down	2.57E–03	5	5	2

*^a^*Betweenness Centrality represents the gene's mediate ability, and Degree represents the number of gene–gene interactions.

*^b,c^*In and out (Indegree and Outdegree) represent the gene regulation numbers upstream and downstream.

### Macrophage proteomics analysis

We used the 2D difference gel electrophoresis (2D-DIGE) method to compare total protein extracts from uninfected macrophages and cells infected with M28 or M5-90 respectively. Biological Variation Analysis (BVA) provides the average ratios between three samples with a threshold of 1.3 and a *t* test confidence level of *P*<0.05, generating a list of spots of interest. All selected spots were picked, digested and identified by MALDI-TOF-TOF. Using BVA software, 17 spots were found to be significantly differentially expressed, and 14 were identified as unique proteins. Of the 14 unique proteins, Actb, Ctsb, Ctsd, Ctss, Apoe, Fabp5, Krt1, and Hnrnpf were all upregulated in M28-infected macrophages compared to macrophages infected with the attenuated M5-90 strain. Of the 11 proteins detected by DGE analysis, eight showed similar expression changes in both DGE and 2D-DIGE analysis (Supplemental Table S6). Four of these were related to lysosome metabolism and all were downregulated in infected macrophages compared to controls, but with lower expression levels in macrophages infected with the M28 virulent strain. Of these proteins, cathepsins (Ctsb, Ctss and Ctsd) participate in both caspase-dependent and -independent programmed cell death induced by a variety of stimuli [34], [35], [36], [37], [38]. Glutathione peroxidase (Gpx1) and capping protein muscle Z-line alpha 2 (Capza2) were upregulated in infected macrophages, and with higher expression levels in macrophages infected with the attenuated strain M5-90 than in M28-infected macrophages. Gpx1 functions in the detoxification of hydrogen peroxide and is one of the most important antioxidant enzymes. It has been reported that Gpx1 protects against CD95-induced apoptosis in cultured breast cancer cells and inhibits 5-lipoxygenase in blood cells; overexpression delays endothelial cell growth and increases resistance to toxic challenge [39]. Fatty acid binding protein (Fabp5) was downregulated in infected macrophages. FabPs play roles in fatty acid uptake, transport and metabolism; FabPs expressed by adipocytes and macrophages (FabP4 and FabP5, respectively) play key roles in regulating systemic metabolism and are important mediators of metabolic syndromes in mice [40]. FabPs are also involved in inflammation. LPS-stimulated cytokine and chemokine secretion, as well as Nos2 and Cox2 production, were inhibited in FabP4-deficient macrophages, and this could reflect reduced responsiveness of the IKK–NFκB pathway [41].

### Validation of DGE data by qPCR

**Figure 6:**
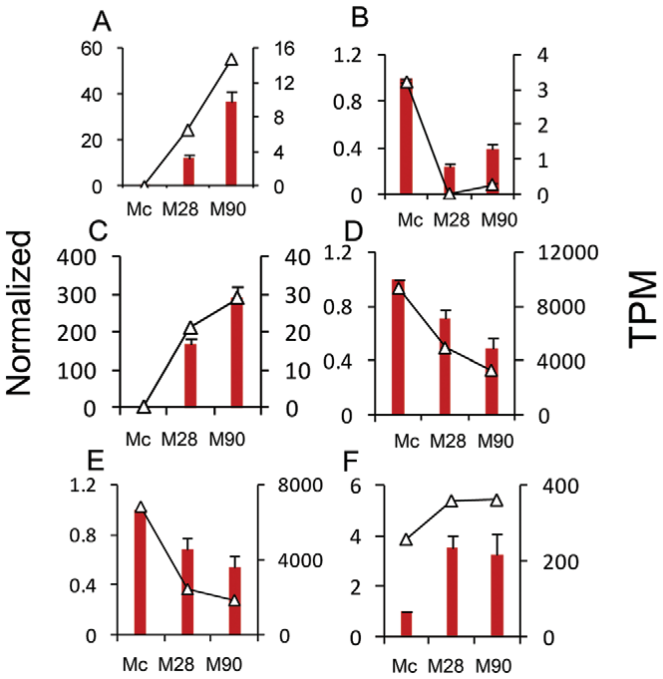
qPCR validation of the DGE data. Relative qPCR was used to measure changes in target gene expression in infected macrophages relative to reference samples. Results are expressed as the target/reference ratio of each sample normalized to the reference gene Fnip1. (A–C). Genes not detected on the 2D-DIGE. Error bars, standard error; TPM, transcripts per million mapped reads.

The *Mus musculus* folliculin interacting protein 1 (Fnip1) RT-PCR product was used as an internal control; expression levels of this gene were unchanged in all samples examined by DGE analysis. To validate the DEGs identified by Solexa sequencing we selected 14 genes for qPCR confirmation. These included nine identified by 2D-DIGE (Hnrnpf, Ctsd, Actb, Capza2, Eef1d, Apoe, Ctsb, Ctss and Fabp5), and five detected by DGE analysis but not by 2D-DIGE. Only Eef1d was discordant between DGE and qPCR analysis. The expression of six genes (slco4a1, B3gnt7, Inhba, Ctss, Ctsd and Capza2) by qPCR fitted the pattern of Tag-seq analysis well (Figure 6). Pearson's correlation coefficient (r) showed that the DGE and qPCR data were highly correlated; genes modulated by *Brucella* infection showed high consistency with r values of ≥0.78 between the two methods. qPCR analysis confirmed the direction of changes detected by DGE analysis, supporting the reliability of the DGE results (Supplemental Table S6).

## Discussion

The M28 virulent strain of *B. melitensis* produces chronic infection whereas the attenuated vaccine strain M5-90 is cleared relatively rapidly from goat, cattle and BALB/c mouse tissues (unpublished data). Mouse macrophages are commonly used in studies of *Brucella* infection. Comparison of responses of BALB/c mouse peritoneal macrophages to infection with strains M5-90 and M28 is likely to cast light not only on common responses to infection but also on differential gene expression in response to infections with *Brucella* strains that differ in virulence. It has was reported earlier that the most significant changes in macrophage gene expression take place early following infection, and global gene expression profiles return to normal between 24 and 48 h post-infection [18]. Indeed, most active brucellacidal activity is thought to occur within 4 h after infection [3], [18], [42]. Accordingly, we used a number of techniques to analyse systematically gene expression changes taking place in mouse macrophages 4 h after infection with two *Brucella* strains of differing virulence.

Here we report the first genome-wide host transcriptional study into different virulent *B. melitensis* infections using the Solexa/Illumina DGE system, a tag-based novel high-throughput transcriptome deep-sequencing method. Previous studies have been limited to commercially available microarrays; the DGE analysis reported here provides a more sensitive and comprehensive unbiased coverage of the entire transcriptome. Because of the nature the DGE methodology we pooled peritoneal macrophages from 10 BALB/c mice to produce macrophage infection samples for deep-sequencing analysis. We achieved a sequencing depth of approximately 3.7 million tags per library (Table 1) and identified over 4000 genes that were differentially expressed following *B. melitensis* infection (Supplemental Table S3). By mapping our DGE tag data onto transcript databases and genomic sequences we were able to show that many alternative and novel transcripts are specifically disease-regulated, information that could not have been obtained by microarray analysis. Our study detected 2117 genes DEGs that overlapped in the Mc versus M28 and Mc versus M5-90 library comparisons. Of the 2105 DGEs exhibiting the parallel changes in both comparisons, the expression levels of 1676 were increased whereas 429 were downregulated. The majority of these overlapping response genes had inflammatory- or apoptosis-related functions, suggesting that macrophages respond similarly to infection with *B. melitensis* strains M28 and M5-90.

Our results confirm previous reports regarding cytokine and chemokine expression during *B. melitensis* infection in mouse macrophages [18], [19] where similar upregulated inflammation-associated genes were observed during *Brucella* infection. For example, the genes encoding nitric oxide synthase 2 (Nos2), interleukin 12a (Il12a), chemokine (C-X-C motif) ligand 2 (Cxcl2), coagulation factor II (thrombin) receptor (F2r) and prostaglandin-endoperoxide synthase 2 (Ptgs2), were significantly upregulated by a factor of ≥eight at 4 h post-infection. Other upregulated genes directly or indirectly related to inflammatory responses included Ccl (2, 3, 4, 5, 7, 9, 22), Ccr7, Cxcl (1,3) interleukins (IL-1a, IL-1b, IL-6), tumor necrosis factor (TNF) and TLR2. The upregulated genes also included Cxcl2 (also known as macrophage inflammatory protein 2-α; MIP2-α), a small cytokine belonging to the CXC chemokine family, growth-regulated protein beta (Gro-β), and Gro oncogene-2 (Gro-2) [43]. In macrophages, inducible nitric oxide synthase (iNOS; Nos2) production has been proposed as a host antimicrobial effector system, displaying activity against fungi, bacteria and parasites [44]. Toll-like receptors (TLRs) recognize *Brucella* spp and bacterial components and initiate mononuclear phagocyte responses that influence both innate and adaptive immunity. Recent studies have revealed the intracellular signaling cascades involved in the TLR-initiated immune response to *Brucella* infection. TLR2 and TLR4 have been implicated in host interactions with *Brucella* [45]. The cell membrane receptor TLR2 recognizes many bacterial, fungal, and viral molecules in addition to particular endogenous substances. In general, this results in the uptake of bound molecules by endosomes/phagosomes and cellular activation. Cytokines participating in this process include TNF-α and diverse interleukins (IL-1α, IL-1β, IL-6, IL-8 and IL-12) [46]. Anti-inflammatory cytokines include interleukin 10 (IL-10), transforming growth factor β (TGFβ1) and IL-1 receptor antagonist (IL-1rn); these cytokines control the damaging effects of pro-inflammatory cytokines and lead to a dynamic balance between these opposing functions [47], [48]. In this study both TGFβ1 and IL-1rn transcripts were upregulated during *Brucella* infection.

Elimination of infected cells via apoptosis (programmed cell death) plays a fundamental role in the defense of multicellular organisms against bacteria, viruses and parasites [49]. Previous studies indicated that smooth *Brucella* spp inhibit apoptosis in macrophages [18], [50], [51], [52], [53]. Our data show that apoptosis-related host genes are similarly up- or downregulated at an early stage of infection in the two infection libraries (Supplemental Table S7). Some proapoptotic molecules, including cyc1 (cyto c), caspase-2 (Casp2), Txnip and Casp7, Cebpb, Eaf2, were significantly down-/upregulated after infection with *Brucella*, and this could result in apoptosis of infected cells. Previous work indicated that smooth virulent *Brucella app* downregulates Casp2 transcriptional levels at an early stage of infection [18], [54] and inhibition of Casp2 activity also increased the number of rough *Brucella abortus* surviving inside macrophages [55]. We have also observed down-/upregulation of the expression of antiapoptotic genes including Aif1, Mkl1, Naip1, Naip6, Birc5, Cited2 and Adam17, Cflar, Pim2, Vegfa. Proapoptotic and antiapoptotic effects taking place concurrently following *Brucella* infection may reflect a balance between apoptotic and antiapoptotic mechanisms; this could represent a sophisticated strategy evolved by *Brucella* to modulate the host cell-death program to promote their own survival.

DEG analysis of the macrophage transcriptional response 4 h after infection with *B. melitensis* strains M28 or M5-90 revealed many macrophage genes whose expression levels changed significantly versus uninfected macrophages. Comparison of the two infection libraries revealed 1019 DEGs whose expression changed in parallel in response to infection; these provide insights into the macrophage antimicrobial defense mechanisms operating in response *B. melitensis* infection (Supplemental Table S3).

Phagocytic cells are known to initiate several different antimicrobial defense mechanisms in response to bacterial infection; these include the generation of oxygen radicals, the extreme acidification of pathogen-containing phagosomes, and fusion of phagosomes with lysosomes, thereby promoting pathogen killing by defensins or degradation by lysosomal enzymes [56]. One study indicated that *Brucella* enters host cells and translocates to the endoplasmic reticulum (ER), a replicative niche, within a few hours after infection, although the majority of bacteria are thought to be killed by phagosome–lysosome fusion [42]. Enrichment analysis of the DEG pathways revealed the lysosome pathway as the most significant. Most lysosome pathway proteins were downregulated in both infection libraries. Of the downregulated lysosome pathway proteins, lysosomal proteases Ctss and Ctsd were also identified by 2D-DIGE and with comparable changes in expression levels. This indicates that lysosome digestive activity is downregulated following infection with *Brucella*. Comparison of the two infection libraries revealed 25 of the 138 genes with lysosome pathway annotations showed increased or decreased expression (Figure S3). The vacuolar-type proton ATPases (Atp6ap1, Atp6v0b and Atp6v0d2), some lysosomal acid hydrolases (glycosidase Neu1 and lipase Lipa), other lysosomal enzymes and activators Cln1 (Ppt1, 2), and lysosome membrane proteins (Endolyn and Cln5), were upregulated. In the lysosomal pathway the V-ATPases, which were altered in expression level, are located in late endosomes and lysosomes and are essential for the acidification of intracellular organelles by coupling ATP hydrolysis to transmembrane proton transport [57]. In mouse macrophages, vacuoles containing live *Brucella suis* are rapidly acidified to a pH of ∼4.0–4.5 at 1 h after infection, and are maintained at this level for at least 5 h; early acidification was found to be essential for the replication of bacteria within macrophages [56]. Compared to uninfected macrophages, the overwhelming majority of DEGs annotated in the lysosome pathway were downregulated. However, downregulation of V-ATPases, some lysosomal acid hydrolases and lysosome membrane proteins was significantly greater in M28-infected cells than in M5-90-infected cells. This indicates that both strains M28 and M5-90 inhibit bacterial killing by host lysosomes, but virulent strain M28 does so more efficiently, in accordance with a previous report that vacuoles containing *Brucella* strain M28 can avoid phagosome–lysosome fusion, thereby promoting access to the replicative niche in the ER [3].

Interaction analysis (Path-Net) of the significant pathways in M28 versus M5-90 (*P*<0.05), comparisons revealed interactions between the significant pathways (Figure 4). Path-Net identified the energy metabolism network (centered on the TCA cycle) and the cell apoptosis signal transduction network (centered on the MAPK signaling pathway). A further small net included three pathways related to glycan metabolism. Smooth *Brucella* inhibited host cell apoptosis by the action of *O*-linked polysaccharides through a TNF-α independent mechanism that is absent from rough *Brucella* [1]. The *O*-linked chains can mask other surface antigens such as outer membrane proteins (OMPs) [58] and are necessary for the full virulence of smooth strains [59]. In recent work the production of proinflammatory cytokines by monocytes/macrophages and dendritic cells was shown to be induced by *Brucella* lipoproteins rather than lipopolysaccharide (LPS) [60], [61], [62]. One study indicated that genes involved in lipid metabolism that were either up- or downregulated 4 h post-infection, and this could reflect macrophage responses to bacterial lipid components [18]. In the energy metabolism localized network, fatty acid metabolism and fatty acid elongation in mitochondria were both significant pathways in M28 versus M5-90. The glycan metabolism-associated significant pathways were glycosylphosphatidylinositol (GPI)-anchor biosynthesis and other glycan degradation pathways. In our previous 2D-DIGE study, analysis of attenuated strain M5-90 cultured on TSB plates showed downregulated expression of OMPs Omp25 and Omp31 (unpublished data). The *Brucella* spp Omp25/Omp31 family comprises seven homologous OMPs, with Omp25 and Omp31 being the major OMPs [63]. Regarding virulence, mutant *B. melitensis*, *Brucella abortus* and *Brucella ovis* strains harboring an inactivated *omp25* gene were found to be attenuated in mice, goats (*B. melitensis*) and cattle (*B. abortus*) [64], [65], [66]. Members of the Omp25/Omp31 family of surface proteins were previously shown to participate in the virulence of some *Brucella* species, and the different distribution of the seven proteins of this family among species could be related to differences in pathogenicity and host preference [67].

In mammals, three major MAPK pathways have been identified: MAPK/ERK, SAPK/JNK and p38 MAPK [68]. MAPK pathways constitute a large kinase network that regulate a variety of physiological processes including cell growth, proliferation, and survival/apoptosis [69]. It is notable that ERK pathway activity is suppressed by JNK/p38 kinases during the induction of apoptosis [68]. A previous study demonstrated that infection with virulent smooth *Brucella* inhibits the activation of the ERK1/2 and p38 MAPK pathways, thus preventing the synthesis of immune mediators that regulate host defense [70].

In the present study, 4 h following infection with either virulent *B. melitensis* M28 or the attenuated strain M5-90, most MAPK signaling pathway genes displayed similar changes in expression. With the exception of ERK (Mapk3), MSK1/2 (Rps6ka4) and TAO1/2 (Taok3), most such genes showed reduced expression in M28-infected cells versus M5-90-infected cells. To identify potential key control genes, proteins of the MAPK (ERK, JNK and p38), Jak–STAT and TLR signaling pathways, in addition to the renal cell carcinoma and related pathways, were built into the protein–protein interaction networks (Signal-Net). Apoptosis-related genes Mapk3, PTPN6, Kras, TRAF6, IKBKE, Chuk, and RelB, were identified as the key genes in Signal-Net (Table 3). Of these key genes, proapoptotic genes in were upregulated more greatly in macrophages infected by strain M5-90 than by M28, whereas expression of the antiapoptotic genes was reduced following M5-90 infection versus M28 infection.

PTPN6 (SHP-1) is an important protein of the Jak–STAT signaling pathway and is located upstream of the MAPK signaling pathway. Extensive studies on PTPN6 revealed that the expression of PTPN6 was diminished or abolished in most cancer cell lines and tissues examined [71]. Dysfunction in PTPN6 regulation can cause abnormal cell proliferation and induce different kinds of cancers [72], [73]. These data suggest that PTPN6 protein plays a switching role in signal transduction pathway regulation; this is supported by the suggestion that SHP-1 can function as a tumor suppressor [74]. In the Signal-net center, ERK1/2 (Mapk3) was inhibited by many upregulated proteins. Msk-1 (Rps6ka4) is an important ERK-activated mediator of mitogen-induced *c-fos* activation, and *c-fos* is a cellular proto-oncogene belonging to the immediate early gene family of transcription factors [75], [76]. The renal cell carcinoma pathway protein Kras is a GTPase and an early player in many signal transduction pathways. In colorectal cancer (CRC), increased Kras 4B expression is accompanied by increased proliferation, decreased apoptosis and activation of MAPK pathways [77].

Previous studies reported that Chuk (IKK-α) is a multifunctional protein which plays a crucial role in the suppression of skin cancer [78], [79], [80]. Chuk indirectly modulates the transcription factor RelB. One study demonstrated that RelB protein contributes to the quality of cell signaling in thymocytes by providing antiapoptotic signals [81]. A recent study revealed that RelB plays an important role in prostate cancer growth *in vivo*, suggesting that the NF-κB alternative pathway contributes to the progression of prostate cancer [82]. Another study indicated that NF-κB pathway activation is crucial for macrophage cell death induced by rough *Brucella* infection [54].

TNF receptor-associated factor 6 (TRAF6), which mediates activation of downstream signaling via NF-κB transcription factors and MAPK cascade-activated AP-1 transcription factors, binds to the type I TGF-β receptor and, upon TGF-β stimulation, causes activation of TGF-β-associated kinase 1 (TAK1) [83]. Activated TAK1 in turn activates MKK3/6, which activates the p38-pathway, leading to apoptosis [84]. TRAF6 of the p38 pathway mediates activation of the Toll-like pathway through interferon regulatory factor 7 (IRF7). The Toll-like pathway protein IKBKE (IKKε), which is reportedly amplified and overexpressed in breast cancer cell lines and patient-derived tumors, similarly suppresses IKKε expression in breast cancer cell lines that harbor IKKε amplifications, inducing cell death [85].

In conclusion, the present study has evaluated the macrophage transcriptome during infection with *B. melitensis* strains M28 and M5-90. By employing a deep-sequencing approach for DEG analysis many more DEGs were detected than using traditional methods, providing new insights into genes involved in host–pathogen interactions. Common changes in gene expression were observed among M28 and M5-90-infected macrophages, suggesting that similar strategies are employed for their survival via the induction of anti-inflammatory and antiapoptotic responses. Numerous genes were found to be differentially expressed between macrophages infected with the virulent and attenuated *B. melitensis* strains, in particular genes participating in the lysosomal and MAPK pathways. High-enrichment GOs included endocytosis, inflammatory, apoptosis, and transport. Path-Net and Signal-Net analysis identified the MAPK pathway as the key regulatory pathway, and the key DEGs of the significant pathways were apoptosis-related genes. These findings indicate that the attenuated strain M5-90 has a reduced ability to avoid phagosome–lysosome fusion and activate MAPK pathways, reflecting the differing intracellular survival abilities of the two strains. Future studies will aid our understanding of the *Brucella* attenuation mechanism by evaluating altered expression of host genes after genetically modified virulent *Brucella* spp infection.

## Supporting Information

Figure S1 **Sequencing saturation analysis.** Saturation analysis of the capacity of libraries showed that the number of detected genes was gradually reduced with increased total sequence tags, when the number of sequencing tags was sufficient. (A) Mc library; (B) M28 library; (C) M5-90 library.
(TIF)

Figure S2 **Distribution of the ratio of distinct tag copy numbers between any two libraries.** The number of distinct tags identified within five is approximately 99.05% of total distinct tags between any two libraries. (A) Library Mc vs. M28; (B) Mc vs. M5-90; (C) M5-90 vs. M28.
(TIF)

Figure S3 **The significant lysosome pathway in the M28 vs. M-90 library (p = 3.73E−09).** 25 out of 138 genes with lysosome pathway annotation showed increased or decreased expression. Red and green represent up-/down-regulated genes.
(TIF)

Table S1 **Summary of gene expression annotation.**
(XLSX)

Table S2 **Summary of antisense gene expression.**
(XLSX)

Table S3 **DiffGeneExpFilter (FDR≤0.001 and |log2Ratio|≥1).**
(XLSX)

Table S4 **Cluster analysis of the intersection of DGEs.**
(XLSX)

Table S5 **Pathway enrichment analysis.**
(XLSX)

Table S6 **Validation of DGE data (2D-DIGE and qPCR).**
(XLSX)

Table S7 **Inflammatory and apoptosis-related DGEs.**
(XLSX)
