# Supplementary figures and images for: Deep-Sequencing Analysis of the Mouse Transcriptome Response to Infection with Brucella melitensis Strains of Differing Virulence
Source: PLoS One. 2011 Dec 28;6(12):e28485. doi: 10.1371/journal.pone.0028485 (PMC3247208; doi:10.1371/journal.pone.0028485)

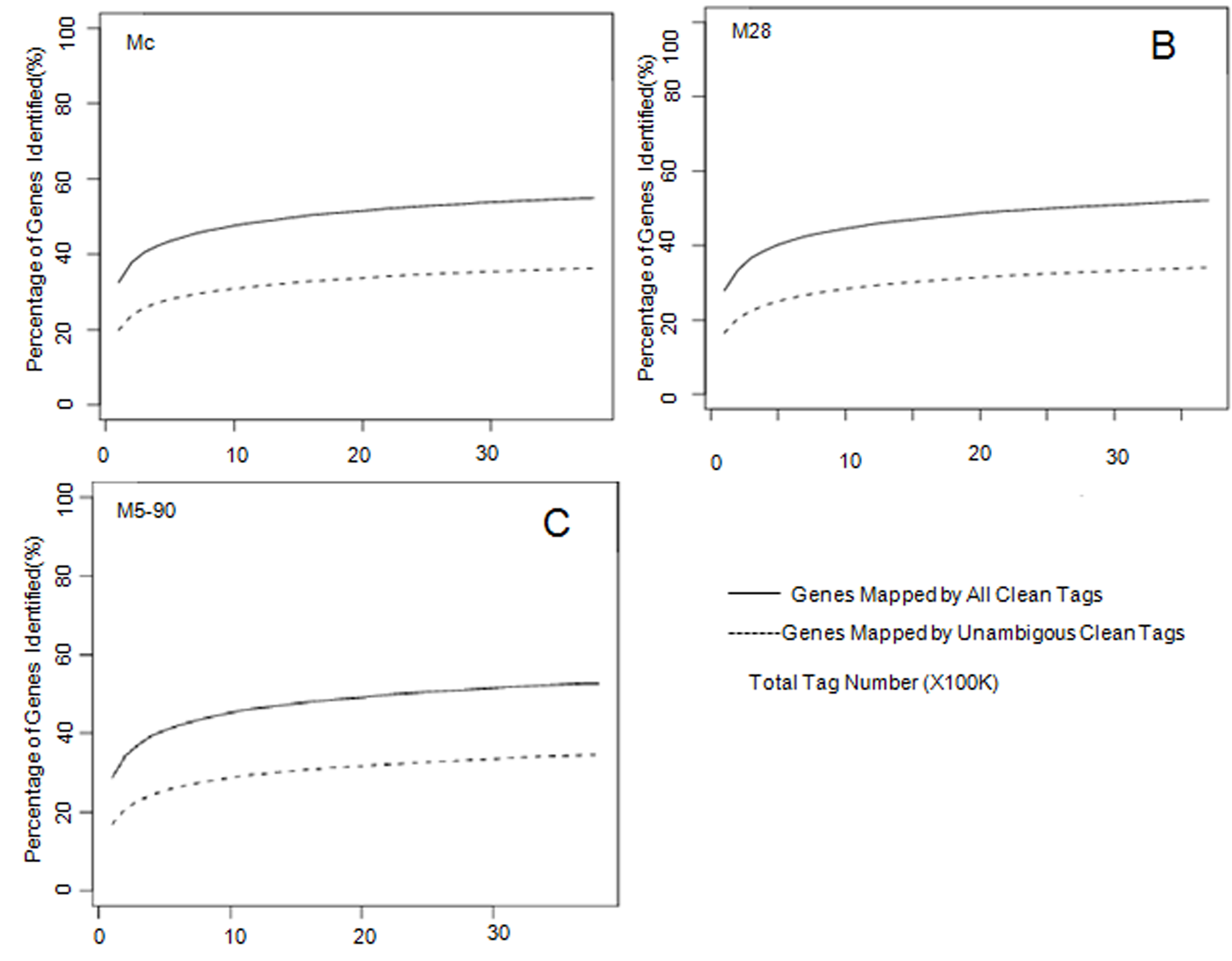

Supplement: Figure S1 — Sequencing saturation analysis. Saturation analysis of the capacity of libraries showed that the number of detected genes was gradually reduced with increased total sequence tags, when the number of sequencing tags was sufficient. (A) Mc library; (B) M28 library; (C) M5-90 library. (TIF) [file pone.0028485.s001.tif]

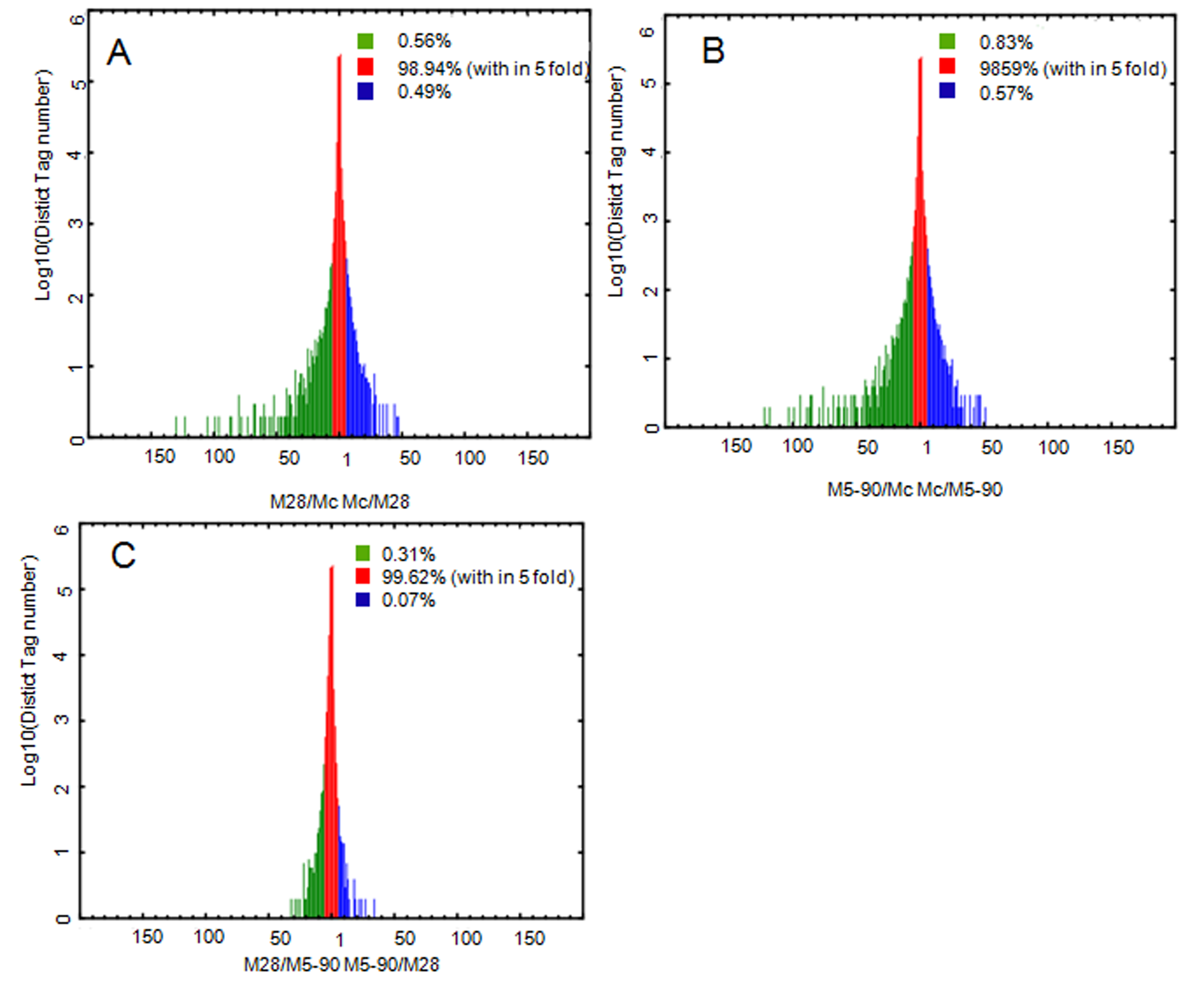

Supplement: Figure S2 — Distribution of the ratio of distinct tag copy numbers between any two libraries. The number of distinct tags identified within five is approximately 99.05% of total distinct tags between any two libraries. (A) Library Mc vs. M28; (B) Mc vs. M5-90; (C) M5-90 vs. M28. (TIF) [file pone.0028485.s002.tif]

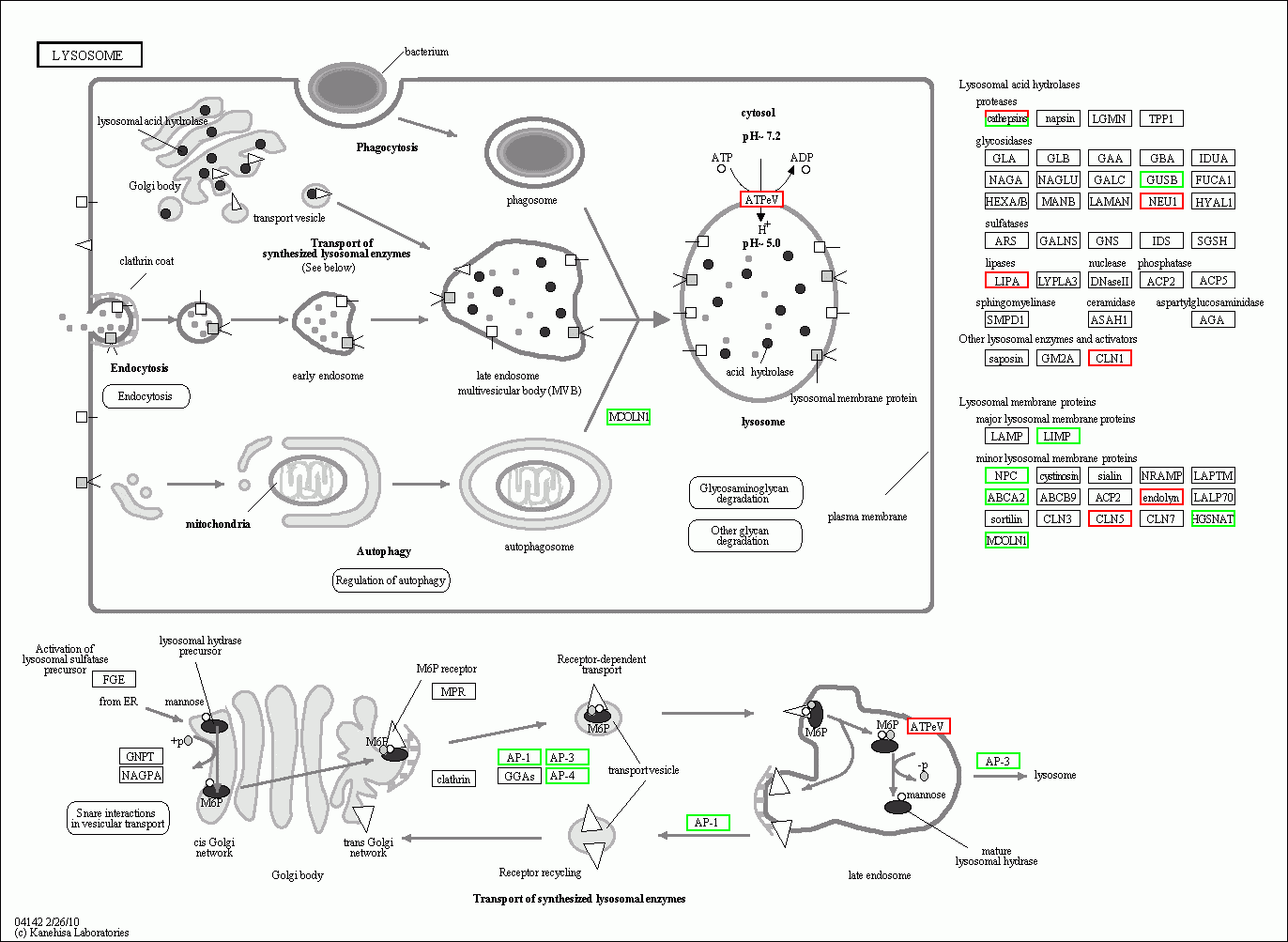

Supplement: Figure S3 — The significant lysosome pathway in the M28 vs. M-90 library (p = 3.73E−09). 25 out of 138 genes with lysosome pathway annotation showed increased or decreased expression. Red and green represent up-/down-regulated genes. (TIF) [file pone.0028485.s003.tif]
